# Identifying Mild Hepatic Encephalopathy Based on Multi-Layer Modular Algorithm and Machine Learning

**DOI:** 10.3389/fnins.2020.627062

**Published:** 2021-01-11

**Authors:** Gaoyan Zhang, Yuexuan Li, Xiaodong Zhang, Lixiang Huang, Yue Cheng, Wen Shen

**Affiliations:** ^1^College of Intelligence and Computing, Tianjin Key Lab of Cognitive Computing and Application, Tianjin University, Tianjin, China; ^2^Department of Radiology, Tianjin First Central Hospital, Tianjin, China

**Keywords:** mild hepatic encephalopathy, dynamic graph properties, multi-layer modular algorithm, disjointness, machine learning, individual discrimination, functional MRI, brain network evolution

## Abstract

Hepatic encephalopathy (HE) is a neurocognitive dysfunction based on metabolic disorders caused by severe liver disease, which has a high one-year mortality. Mild hepatic encephalopathy (MHE) has a high risk of converting to overt HE, and thus the accurate identification of MHE from cirrhosis with no HE (noHE) is of great significance in reducing mortality. Previously, most studies focused on studying abnormality in the static brain networks of MHE to find biomarkers. In this study, we aimed to use multi-layer modular algorithm to study abnormality in dynamic graph properties of brain network in MHE patients and construct a machine learning model to identify individual MHE from noHE. Here, a time length of 500-second resting-state functional MRI data were collected from 41 healthy subjects, 32 noHE patients and 30 MHE patients. Multi-layer modular algorithm was performed on dynamic brain functional connectivity graph. The connection-stability score was used to characterize the loyalty in each brain network module. Nodal flexibility, cohesion and disjointness were calculated to describe how the node changes the network affiliation across time. Results show that significant differences between MHE and noHE were found merely in nodal disjointness in higher cognitive network modules (ventral attention, fronto-parietal, default mode networks) and these abnormalities were associated with the decline in patients’ attention and visual memory function evaluated by Digit Symbol Test. Finally, feature extraction from node disjointness with the support vector machine classifier showed an accuracy of 88.71% in discrimination of MHE from noHE, which was verified by different window sizes, modular partition parameters and machine learning parameters. All these results show that abnormal nodal disjointness in higher cognitive networks during brain network evolution can be seemed as a biomarker for identification of MHE, which help us understand the disease mechanism of MHE at a fine scale.

## Introduction

Hepatic encephalopathy (HE) is a syndrome of central nervous system dysfunction based on metabolic disorders caused by severe liver disease (Agrawal et al., 2015; Zhang et al., 2017b). Mild hepatic encephalopathy (MHE), as a mildest form of HE spectrum, has no recognizable clinical symptoms of HE, but is characterized by subtle neurocognitive and psychomotor deficits, such as psychomotor slowing, shortened attention concentration, dysfunctional executive abilities, and memory loss (Agrawal et al., 2015). MHE impairs individual’s daily functioning, driving performance, work performance and learning ability (Agrawal et al., 2015). MHE has a probability of 40% converting to overt HE within six months if not treated promptly, and once converted, patients will have increased falls, short survival and high mortality (Campagna et al., 2014; Zhang et al., 2018b). Early diagnosis and effective treatment are essential to reduce conversion to overt HE and to improve patients’ quality of life. Currently, it is difficult to clinically diagnose MHE patients from cirrhotic patients with no HE (noHE). Therefore, it is of great significance to understand the dysfunction mechanism of MHE and to explore the biomarkers for precisely clinical diagnosis.

In the past years, resting state-functional magnetic resonance imaging (RS-fMRI) characterized by non-invasiveness, high sensitivity, ultra-fast imaging and no requirement of engaging in a task, has attracted more and more attention in the study of hepatic encephalopathy (Zhang et al., 2014a, 2017a). It measures the relative changes of blood oxygen level dependent (BOLD) signals as a representation of spontaneous neural activities in the human brain. A number of studies have explored changes in brain functional network that are related to cognitive function in patients with MHE. Zhang et al. (2014b) used graph theory analysis and found that changes in small-world property in patients with MHE were related to their cognitive impairment. Qi et al. (2012) used the independent component analysis (ICA) to evaluate the difference of resting state networks between MHE patients and healthy controls (HCs), revealing that MHE patients showed significantly decreased functional connectivity in dorsal attention network (DAN), both decreased and increased functional connectivity in default mode network (DMN), auditory network (AN) and visual network (VN). No significant differences were found in self-referential network (SFN) and sensorimotor network (SMN) between MHE and HCs. Regional homogeneity (ReHo) analysis of resting state brain activity showed that compared with the noHE patients, the MHE patients show decreased ReHo value in the bilateral parietal lobes including the precuneus, supplementary motor area, frontal lobes and occipital lobes including the cuneus. With whole-brain functional connectivity analysis, a study (Zhang et al., 2012) concluded that compared with HCs, MHE patients presented widespread cortical and subcortical functional connectivity alterations that were correlated with neuropsychologic impairment. Particularly, impairment in the basal ganglia-thalamocortical circuit may play a key role in mediating neurocognitive dysfunction, especially the psychomotor speed and attention deficits in MHE patients. These studies indicate that changes in resting state functional connectivity can reflect abnormal cognitive function in MHE patients. Some other studies combined the resting state brain activity or functional connectivity features with machine learning method (i.e. support vector machine, linear discriminant analysis) to investigate the early identification of MHE (Chen et al., 2014, 2016a,b; Jiao et al., 2017). However, the discrimination accuracy is not satisfying to meet the clinical demands. Moreover, these previous studies assumed the brain as a static functional connectivity pattern during the whole resting state scan (at least 5 min) and ignore the fact that the human brain is obviously a dynamically interactive system, and even at the relatively sluggish temporal resolution of fMRI (Cai et al., 2019).

Recently, dynamic functional connectivity analysis has drawn more and more attention in studies of brain disease (Hutchison et al., 2013; Calhoun et al., 2014; Kucyi and Davis, 2015), which can capture transient functional connectivity changes and describe dysfunction of MHE at a fine scale. For example, abnormality in dynamic brain function has been observed in autism spectrum disorder (Harlalka et al., 2019), and epilepsy (Tailby et al., 2018). Dynamic graph analysis is a promising avenue to quantitatively characterize the time evolving brain dynamics at a system level. It assumes the whole brain functional connectivity as a graph, there is a modular structure in the brain network graph (Rubinov and Sporns, 2010) and the modular structure evolves dynamically across time. It was reported that the modularity of dynamic functional connectivity networks can change on a very short time scale, and thus this approach may be able to track transient changes in functional connections between brain regions (Betzel et al., 2016). Bassett et al. (2011) and Cole et al. (2013) used multi-layer modular analysis on the dynamic graph structure of the brain imaging data and found that the modular structure in the dynamic network was able to represent the cognitive function. Therefore, we believe that dynamic graph analysis will give us a deeper insights into the abnormal cognitive function of MHE.

In this study, we intended to use a multi-layer modular analysis method to detect the changes of dynamic graph properties of brain network in patients with MHE, investigate the clinical correlation of these properties and further construct a machine learning model based on the selected network properties to identify MHE from noHE at the individual level. We hypothesized that dynamic brain connectivity analysis can reveal the complex, adaptive, cognitive dysfunction underlying MHE and the temporal variation of the brain network metrics could provide rich diagnostic information to discriminate MHE from noHE.

## Materials and Methods

### Participants

A total of 103 participants was used in this study, including 30 MHE patients, 32 noHE patients and 41 HCs, see Table 1. HC group was added to study dysfunction mechanism as a contrast. The machine learning model was performed on the patient groups because the discrimination of cirrhosis with MHE from that with no HE is the main concern of clinical doctors. This study was approved by the Medical Research Ethics Committee of Tianjin First Central Hospital. Written informed consent was obtained from each subject prior to participation in this study.

**TABLE 1:** Demographic, neuropsychological and clinical data.

Protocols	HC (*n* = 41)	noHE (*n* = 32)	MHE (*n* = 30)	*p*-value	χ^2^/F/T value
Sex (M/F)	28/13	18/14	21/9	0.449^*a*^	1.603^*a*^
Age (years)	50.1 ± 7.3	47.9 ± 8.2	50.9 ± 6.3	0.153^*b*^	1.917^*b*^
Education (years)	13.0 ± 2.6	12.3 ± 3.5	12.3 ± 3.0	0.499^*b*^	0.699^*b*^
NCT-A (seconds)	41.6 ± 3.2	43.1 ± 12.7	78.9 ± 14.6	< 0.001^*b*^	93.427^*b*^
	–	–	–	< 0.001^*c*1^	10.306^*c*1^
	–	–	–	< 0.001^*c*2^	12.011^*c*2^
	–	–	–	0.599^*c*3^	0.528^*c*3^
DST (score)	47.9 ± 10.2	43.1 ± 9.5	30.1 ± 11.7	< 0.001^*b*^	25.920^*b*^
				< 0.001^*c*1^	−4.792^*c*1^
	–	–	–	< 0.001^*c*2^	−6.685^*c*2^
	–	–	–	0.043^*c*3^	−2.071^*c*3^
Prothrombin time (seconds)	–	16.8 ± 6.0	18.8 ± 5.2	0.105^*c*1^	1.353^*c*1^
Albumin (mg/dl)	–	32.0 ± 5.8	30.0 ± 5.9	0.565^*c*1^	−1.355^*c*1^
Total bilirubin (mg/dl)	–	96.1 ± 139.3	106.4 ± 170	0.737^*c*1^	0.268^*c*1^
Blood ammonia (μmol/L)	–	55.1 ± 21.3	72.6 ± 31.5	0.009^*c*1^	2.543^*c*1^
Child-Pugh A/B/C	–	5/15/11*	1/7/22	–	–

Data are presented as mean ± standard deviation. * : One person lacked information. a: Pearson χ^2^ test of three groups (two-tailed), b: One-way analysis of variance test among three groups (two-tailed), c1: Two-sample t test between MHE and noHE groups (two-tailed), c2: Two-sample t test between MHE and HC groups (two-tailed), c3: Two-sample t test between noHE and HC groups (two-tailed). DST, digit-symbol test; HC, healthy control; MHE, mild hepatic encephalopathy; NCT-A, number connection test of type A; noHE, cirrhotic patients without clinical hepatic encephalopathy.

Before functional magnetic resonance imaging (fMRI) scanning, patients with cirrhosis were tested for blood ammonia, prothrombin time, total bilirubin, and albumin biochemical parameters to assess liver function (Table 1). Functional status of cirrhosis was assessed by child-pugh score (Pugh et al., 1973). The HC group had no liver or other systemic problems, no history of psychosis or neuropathy.

As recommended by previous studies (Weissenborn et al., 2001; Li et al., 2013), neuropsychological tests including Number Connectivity Test A (NCT-A) and Digit Symbol Test (DST) were performed on all subjects to diagnose MHE by clinic. To be specific, linear regression models of NCT-A and DST were estimated with regressors of age and education in the HC group, and then the model was used to predict scores of NCT-A and DST for subjects in patient groups. The difference between the predicted value and the true value was calculated, and the patient with either DST or NCT-A difference greater than 2 standard deviation was determined as MHE.

In addition, some patients used antibiotics if they have infections, such as spontaneous bacterial peritonitis, and pulmonary infection. Lactulose was used in 19 patients to improve feces excretion function, and they took 5–10 g lactulose three times a day. Patients were excluded if they took psychotropic medications, suffered from uncontrolled endocrine disorders, had other neuropsychiatric disorders or metabolic diseases, had alcohol abuse within 6 months prior to the study, or had large head motions during scanning. In the end, the aforementioned 103 participants were remained.

### Overview of Methodology

**FIGURE 1:**
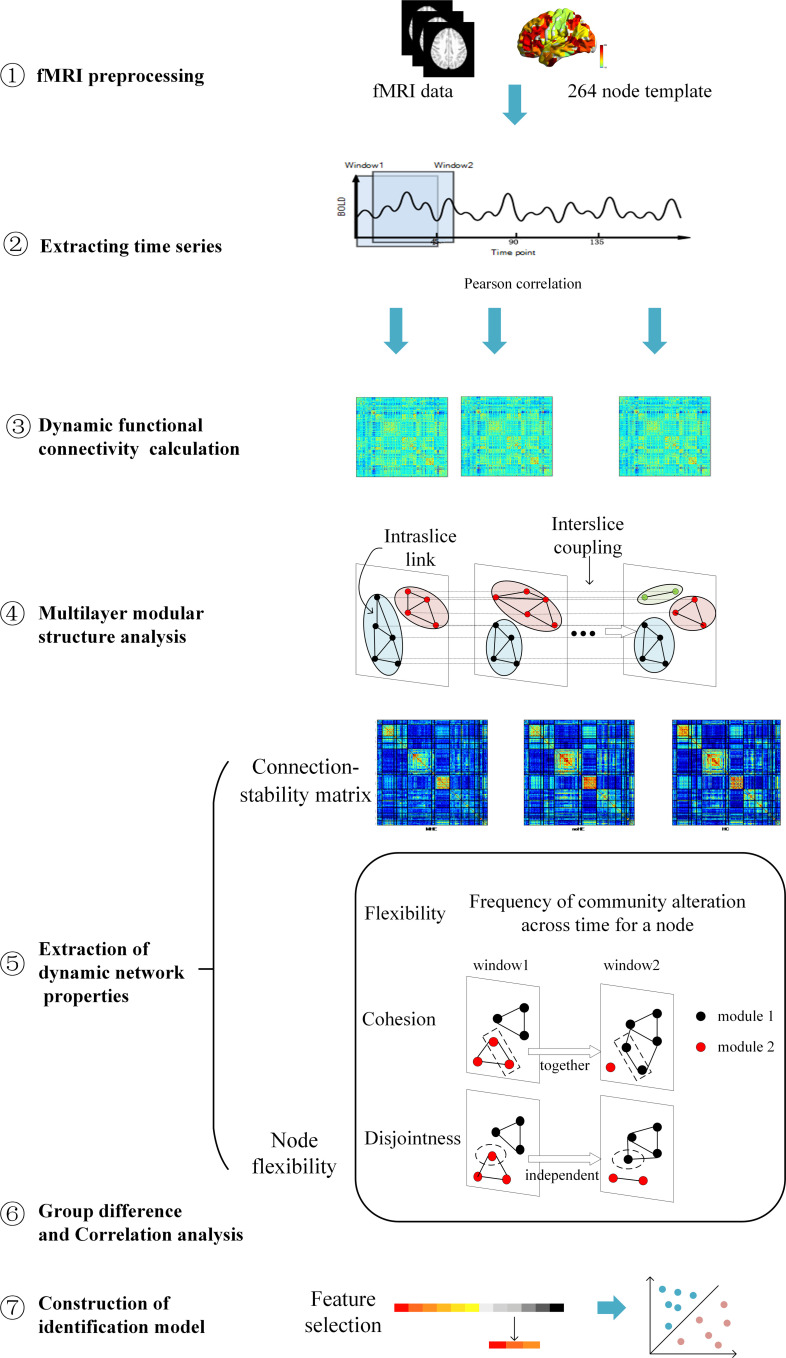
An overview of data analysis pipeline. **➀**. fMRI data preprocessing; **➁**. Extracting regional time series based on Power’s 264 atlas; ➂. Sliding-window based dynamic functional connectivity matrix calculation; **➃**. Multi-layer modular structure calculation from the dynamic brain connectivity matrix; **➄**. Extraction of dynamic network properties; **➅**. Group difference of dynamic network properties and their correlations with clinical cognitive test scores; **➆**. Construction of identification model based on dynamical network properties for individual MHE discrimination.

An overview of the framework is summarized in Figure 1. First, resting-state fMRI data were preprocessed. Second, the nodal time series were extracted using a sliding time window to calculate the dynamic functional connection graph. Third, the dynamic functional connection matrix is constructed with Pearson correlation and these matrix can be seemed as a dynamic graph. Fourth, the multi-layer modularization algorithm is used to determine the temporal module structure in the dynamic graph. Fifth, several dynamic graph properties describing brain connection stability and node changes of module affiliation during brain network evolution over time were calculated. Sixth, inter-group difference of these metrics and their correlations with the neuropsychological and clinical test scores were performed at two levels of local network and individual node. Finally, the metrics with largest inter-group differences were selected as features to identify individual MHE from noHE by machine learning, and discriminant analysis were used to explore the contribution of each feature.

### MRI Scanning Parameters

The MRI data were collected using a Siemens 3.0T (TIM-Trio, Siemens Medical Solutions, Erlangen, Germany) MRI scanner with a 32-channel head coil. Foam padding was used to reduce head motion. The scan sequences of each subject included conventional T2WI, 3D-T1WI and resting-state fMRI. Two-dimensional T2-weighted turbo spin echo (TSE) and T1-weighted MPRAGE sequences were used to detect brain lesions. Gradient echo plane imaging (EPI) sequence was used to obtain BOLD image with the following parameters: measurement = 200, echo time (TE) = 30ms, repeat time (TR) = 2500 ms, flip angle (FA) = 90°, field of view (FOV) = 220 mm × 220 mm, matrix = 96 × 96, slice thickness = 3 mm, slice gap = 0.3 mm, and number of slices = 40. The total scan time of resting-state data was 500 s. The subjects were asked to close their eyes, relax physically and mentally, stay awake, keep their heads and bodies still, and try not to think about anything.

### Data Preprocessing

The fMRI images were preprocessed using MATLAB 2013a (MathWorks, Natick, MA, United States) with the GRETNA software package^1^. The first 10 time points were removed to equalize the magnetization and to allow subjects to adapt to the scanning environment. Time slice correction and head motion correction were performed for the remaining 190 time points. We also calculate the frame-wise displacement (FD), which represents the volume changes of the head position. Subjects were removed with mean FD > 0.2 mm, maximum translation > 2 mm, maximum rotation > 2 degree (Greve and Fischl, 2009). The images were then normalized, including co-registered, segmented, normalized to EPI Spaces and resampled to 3 × 3 × 3 mm^3^, After spatial smoothing of the image with a full width at half maxima (FWHM) kernel of 8 mm, linear trend of BOLD signal was removed and covariates such as white matter signal, cerebrospinal fluid signal (CSF) and 24 head motion parameters were regressed to reduce the influence of other factors. According to previous studies, global signal regression (GSR) has been shown to cause a possible inverse correlation effect in resting brain networks, so GSR is not used in image preprocessing (Murphy et al., 2009; Weissenbacher et al., 2009; Saad et al., 2012). Finally, the regression time series was temporal filtered (0.01–0.1Hz) (Liu and Duyn, 2013).

### Dynamic Function Network Construction

We used a whole brain template to define the brain as 264 nodes (Power et al., 2011). The 14 functional networks were partitioned based on 264 nodes for subsequent network analysis (Power et al., 2013). The mapping between names of the network and their abbreviations, member nodes are provided in Table 2. The Pearson correlation was applied to obtain the whole brain functional connectivity matrix. Then, to track the dynamic changes in the brain over a short period of time, we used a sliding window length of 45TR (112.5s), which has been proved to achieve better identification performance around 110s based on a previous study (Liu et al., 2017), and the window moves with a step size of 1TR (2.5s). The remaining 190 time points were divided into 146 time windows (1-45, 2-46, 3-47…, 146-190). A Fisher Z-Transformation was performed on the connection matrix within each window of the subject for subsequent analysis. Therefore, the dynamic functional connectivity matrix was obtained and it can be viewed as a dynamic graph.

**TABLE 2:** The mapping between network names, their abbreviations and member nodes.

Networkindex	Network name	abbreviation	member node
1	Uncertain	Uncertain	1-12,84-85,132,140-142,182-185,247-250,253-254
2	Sensory_Somatomotor_Hand	SSHN	13-41,255
3	Sensory_Somatomotor_Mouth	SSMN	42-46
4	Cingulo-opercular_Task_Control	CON	47-60
5	Auditory	AN	61-73
6	Default_mode	DMN	74-83,86-131,137,139
7	Memory_retrieval	MRN	133-136,221
8	Ventral_attention	VAN	138,235-242
9	Visual	VN	143-173
10	Fronto-parietal_Task_Control	FPN	174-181,186-202
11	Salience	SN	203-220
12	Subcortical	Subcortical	222-234
13	Cerebellar	Cerebellar	243-246
14	Dorsal_attention	DAN	251-252,256-264

### Multi-Layer Modular Algorithm

A multi-layer modular algorithm was used to determine the modular structure in the dynamic functional connection matrix.

In order to quantify the temporal and spatial interactions of brain nodes, we used an iterative and orderly Louvain algorithm to track the changes of community partition over time. Compared with the Louvain algorithm, the multi-layer modular algorithm adds one parameter called omega, which is used to control the strength of the coupling between time layers. The optimization goal is to maximize the modularity (Q) of the brain network. That is to maximize intra-modular connectivity and minimize inter-modular connectivity, so as to find a stable modular structure (Newman, 2004). The calculation method of multi-layer modularization is as follows:

(1) $$\begin{array}{c} Q\left(\gamma ,\omega \right)=\frac{1}{2\mu }\sum_{ijsr}[\left(A_{ijs}-\gamma_{s}\frac{k_{is}k_{js}}{2m_{s}}\right)\delta \left(s,r\right)+\delta \left(i,j\right)\cdot \omega_{jrs}]\delta \left(M_{is},M_{jr}\right) \end{array}$$

(2) $$\begin{array}{c} m=\frac{1}{2}\sum_{ij}A_{ij} \end{array}$$

(3) $$\begin{array}{c} \mu =\frac{1}{2}\sum_{jr}k_{jr} \end{array}$$

where *A*_*ijs*_ is the correlation between nodes *i* and *j* under the sliding window of time point *s*, *k*_*is*_ is the degree of node *i* under time point *s* and *k*_*is*_*k*_*js*_/2*m*_*s*_ represents the Newman-Girvan null model of intra-network connections. γ_*s*_ is the topological resolution parameter under a time point *s* or layer *s*. ω_*jrs*_ is the time-coupling parameter between node *j* in the time window *r* and node *j* in the time window *s*. For δ(*M*_*is*_,*M*_*jr*_), if node *i* and *j* belong to the same module, it is 1; otherwise, it is 0. For δ(*s*,*r*), if *s* = *r*, it is 1; otherwise, it is 0. For δ(*i*,*j*), if *i* = *j*, it is 1; otherwise, it is 0.

In our study, we used Genlouvain Matlab toolbox (Jutla et al., 2011–2019) to calculate the modular structure of the brain. And the default value 1.0 was chosen as the gamma and omega values. Due to the variability in optimizing the partition, we repeated the algorithm 100 times.

#### Connection-Stability Matrix

In order to study the characteristics of nodal connection that are more stable in dynamic brain interaction, we calculated the connection-stability scores between nodes by dividing the module results. The connection-stability score was calculated as the proportion of time windows in which a given node pair is assigned into the same module, with nodal network membership defined on the basis of multi-layer modular algorithm (gamma = 1.0 and omega = 1.0). If two nodes were assigned to the same module in a time window, the connection-stability score is 1, else it is set 0. A higher value indicates that two nodes were relatively stable participating in the same community. The output is G = N × N matrix, where each element (*m, n*) is connection-stability scores between node *m* and node *n*. To avoid the chance of a result, the final result of each metric was the average of 100 runs.

#### Dynamic Nodal Metrics Extracted From Modular Structure

From modular partition result, we extracted three dynamic metrics to describe the nodal properties, namely nodal flexibility, cohesion, disjointness. These metrics measured the dynamic reconfiguration that occurs in the brain over time, and a higher dynamic metrics would imply a hypervariable connection. To avoid the chance of a result, the final result of each metric was the average of 100 runs.

(1) Flexibility
  Node flexibility is defined as the ratio of the number of times a node changes communities to the number of possible times a node changes communities. It’s a number between 0 and 1 where 1 means that the node is most flexible over time.

(4) $$\begin{array}{c} f_{i}=1-\frac{1}{T-1}\sum_{s=1}^{T-1}\delta \left(G_{i,s},G_{i,s+1}\right) \end{array}$$

For δ(*G*_*i*,*s*_,*G*_*i*,*s* + 1_), if node *i* and *j* belong to the same module, it is 1; otherwise, it is 0. *T* is the total number of time windows.
(2) Cohesion
  Although node flexibility determines how often the node changes the community, it does not describe how the node changes the community. Node cohesion describes how often one node changes a community with another (Telesford et al., 2017). A high cohesion value indicates that the node typically changes the community along with other nodes. A low cohesion value indicates that the node rarely changes the community with other nodes.
(3) Disjointness
  Node disjointness is defined as the ratio of the number of times a node can change a community independently to the number of times a node can change a community (Telesford et al., 2017). It quantify the percentage of times a node changes its community independently. The global disjointness for each subject was calculated as the mean disjointness values of all nodes of each subject.

### Group Differences in Dynamic Graph Metrics

We used Kruskal-Wallis nonparametric one-way analysis of variance (ANOVA) to analyze differences among three groups (MHE, noHE, and HCs) in dynamic indicators and connection-stability. The analyses were performed at two levels of brain network and brain region nodes. If there was a statistical difference, a *post hoc* test were performed to detect the inter-group difference. Significant group differences were tested at *p* < 0.05 after corrections for multiple comparisons.

### Correlation With Neuropsychological Scores

We used partial correlation analysis to examine the relationship between dynamic metrics, connection-stability of each patient and the neuropsychological scores, meanwhile the age, gender, educational level, and head motion parameters were all used as covariates to avoid their influences. Multi-level correlations have been performed, from the network-level and individual node-level. At the network level, the 264 nodes were divided into 14 brain networks (Power et al., 2013).

### Discrimination of Individual MHE From noHE

In this study, *F*-score was used as feature selection method to measure the ability of the dynamic graph metrics (Chen and Lin, 2006). The F score of the *i*th feature is defined as follows:

(5) $$\begin{array}{c} F\left(i\right)=\frac{{\left({\bar{\bar{x}}}_{i}^{\left(+\right)}-{\bar{x}}_{i}\right)}^{2}+{\left({\bar{x}}_{i}^{\left(-\right)}-{\bar{x}}_{i}\right)}^{2}}{\frac{1}{n_{+}-1}\sum_{k=1}^{n_{+}}{\left(x_{k,i}^{\left(+\right)}-{\bar{x}}_{i}^{\left(+\right)}\right)}^{2}+\frac{1}{n_{-}-1}\sum_{k=1}^{n_{-}}{\left(x_{k,i}^{\left(-\right)}-{\bar{x}}_{i}^{\left(-\right)}\right)}^{2}} \end{array}$$

Where the $\bar{x}$ is the average values of all the sample, while $\bar{x}$_*i*_^(+)^ and $\bar{x}$_*i*_^(–)^ represent the mean values of all the positive and negative samples, respectively. *k* represents each instance of the specific *i*th feature. *F*(*i*) calculated the difference of *i*th feature between the two groups. The larger the *F*-score, the stronger the discrimination of this feature. We ranked all the features according to the *F*-score, and selected the top k largest features for the following classification analysis. The k was determined based on the average classification performance of all loops. Finally, 23 features were selected in this study.

Support vector machine (SVM)^2^ was applied to classify individual patient with MHE from the noHE using the extracted features because SVM is especially suitable for fMRI data with small samples and a high dimension. A leave-one-out cross-validation(LOOCV) method was used to estimate the classification performance due to its small-sample friendly nature (Pereira et al., 2009).

To assess the performance of our method, we calculated classification accuracy, sensitivity, and specificity, respectively. Sensitivity measures the proportion of positives that are correctly identified as such. Specificity measures the proportion of negatives that are correctly identified as such. In addition, the receiver operating characteristic (ROC) analysis was used to evaluate the performance of the classifier. The larger area under ROC curve (AUC) indicates a better discriminant power (Fawcett, 2006). We used 1000 permutation tests to determine whether the accuracy of the results were higher than the chance level. Because the LOOCV approach makes the feature selection of the sample subset in each fold slightly different, the discriminant features were defined as the top 23 frequently occurred features in all folds.

### Validation

To validate the robustness of our findings, we also repeated the analysis using multiple parameters. As for the analysis results of correlation and group difference, we mainly verify the robustness of the results under different time windows and different modularization algorithm parameters. For the time window size, a window length of 40TR (100 s), 50TR (125 s) and 55TR (137.5 s) were considered. For multilayer modular parameters, values near gamma = 1.0 and omega = 1.0 were analyzed repeatedly. As for the classification results, the comparisons of feature selection method and classifier kernel function were performed. We used the commonly used relief method (Kira and Rendell, 1992) and the linear kernel SVM classifier as a comparison.

## Results

The results reported in the group comparison and correlation section are based on the parameters (gamma = 1.0; omega = 1.0; window size = 45TR) that can obtain the best identification accuracy. Results based on different parameters for validation were reported in the classification section.

### Effect of Disease on Network Connection-Stability Matrix

**FIGURE 2:**
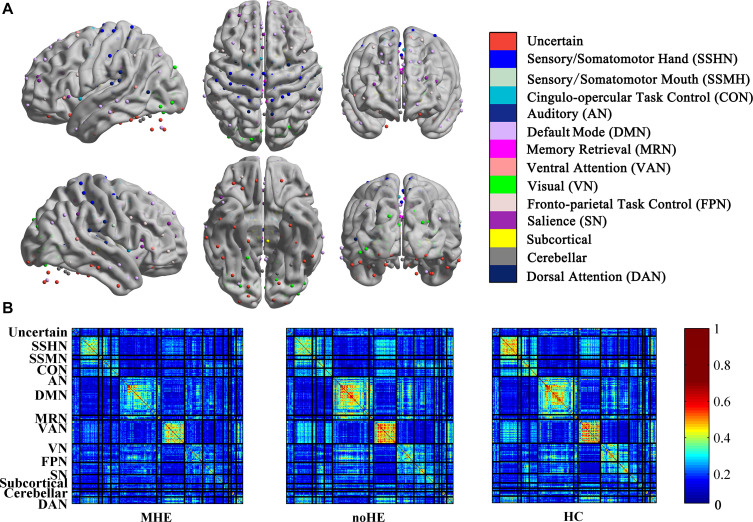
The connection stability profile of the brain network across time in MHE, noHE and HC groups. **(A)** The distribution of 14 brain networks in the 264-node brain template; **(B)** The connection-stability matrix distributed in the 14 networks in MHE, noHE and HC groups. MHE, minimal hepatic encephalopathy; noHE, non-hepatic encephalopathy; HC, health control.

The connection-stability matrix over time for MHE, noHE and HC was shown in Figure 2. Before comparison, the connection stability values were averaged in each network module. One-way ANOVA results indicated that average connection-stability score in sensory/somatomotor mouth network (SSMN), default mode network (DMN), cingulo-opercular task control network (CON), and frontro-parietal task control network (FPN) showed significant differences among the three groups, but only FPN survived false discovery rate (FDR) correction. *Post hoc* analyses were performed to detect the inter-group difference. Results showed that the average connection-stability scores of MHE patients were significantly lower than that of HC in CON and FPN, and marginally significant (adjusted *p* = 0.054) in DMN after FDR correction, but no significant differences were observed between other groups. The *p*-value in each comparison and significances after FDR correction were all listed in Table 3.

**TABLE 3:** The differences in average network connection-stability scores among MHE, noHE and HC groups.

Network	*p* value (ANOVA)	*p* value (MHE vs. HC)	Median MHE	Median noHE	Median HC
Uncertain	0.941	–	0.260	0.259	0.268
SSHN	0.231	–	0.367	0.382	0.406
**SSMN**	**0.042**	**0.039**	0.488	0.492	0.565
**CON**	**0.022**	0.006*	0.333	0.358	0.389
AN	0.32	–	0.349	0.345	0.366
**DMN**	**0.031**	**0.018**	0.307	0.351	0.356
MRN	0.26	–	0.455	0.517	0.502
VAN	0.392	–	0.324	0.333	0.317
VN	0.251	–	0.373	0.465	0.467
**FPN**	0.001*	<0.001*	0.308	0.331	0.389
SAN	0.054	–	0.287	0.355	0.347
Subcortical	0.772	–	0.330	0.324	0.323
Cerebellar	0.45	–	0.493	0.478	0.518
DAN	0.523	–	0.381	0.402	0.365

Results with *p* < 0.05 are bold in the table. * indicates that *p* < 0.05 after FDR corrections for multiple comparisons.

### Correlation Results Between Network Connection-Stability and Clinical Scores

We also calculated the correlation between the average network connection-stability score and the clinical scores in all cirrhotic patients. The results showed a significant positive correlation between DST and network connection-stability scores in DMN, visual network (VN), FPN, salience network (SN), and subcortical network (Table 4). All the results were reported at *p* < 0.05 after corrections for multiple comparisons.

**TABLE 4:** Networks showing significant correlation of connection-stability with clinical test scores.

Network	Blood ammonia[r]	Blood ammonia[p]	NCT[r]	NCT[p]	DST[r]	DST[p]
DMN	−0.12	0.37	−0.217	0.102	0.35	0.007*
VN	−0.202	0.128	−0.132	0.324	0.376	0.004*
FPN	0.004	0.979	−0.278	**0.035**	0.338	0.009*
SN	−0.147	0.272	−0.212	0.111	0.525	<0.001*
Subcortical	−0.053	0.69	−0.106	0.43	0.361	0.005*

Results with *p* < 0.05 are bold in the table. * indicates that *p*-value survived FDR correction.

### Effect of Disease on Dynamic Nodal Metrics

**FIGURE 3:**
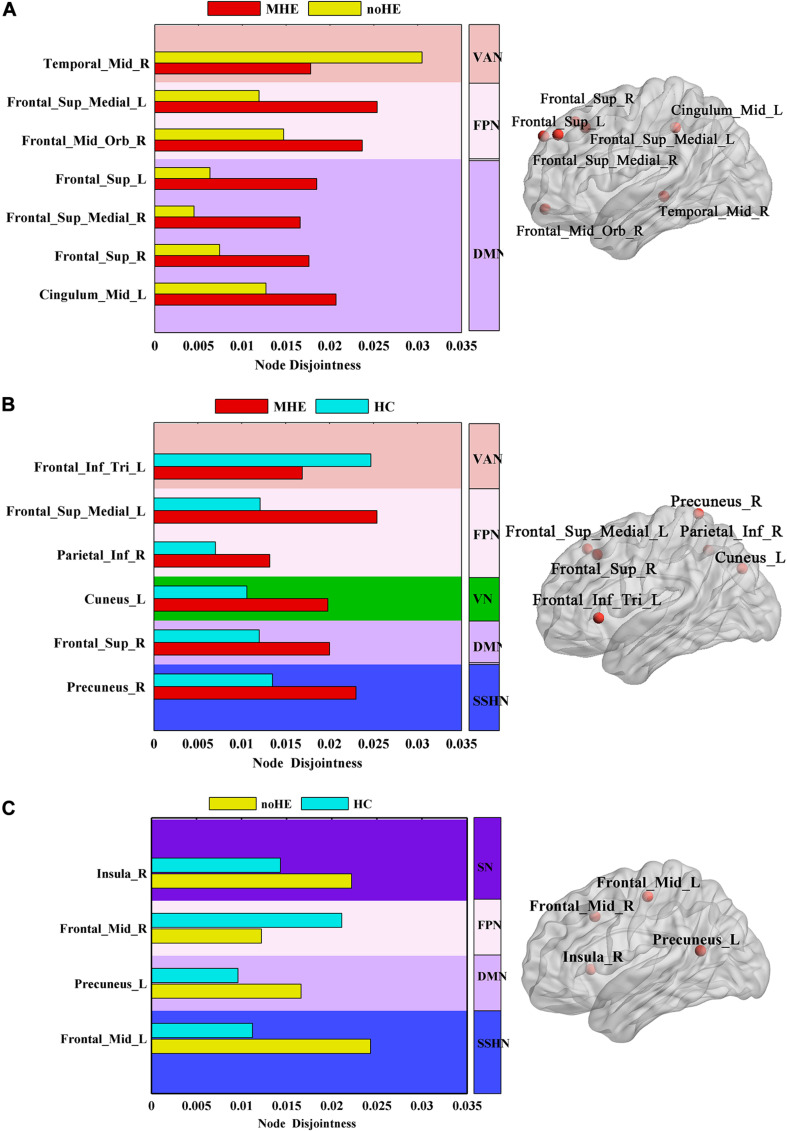
The differences in node disjointness during brain network evolution among the three groups by one-way analysis of variance (ANOVA). **(A–C)** The difference regions of the brain networks and the corresponding spatial locations in *post hoc* comparison of MHE with noHE, MHE with HC and noHE with HC. MHE, minimal hepatic encephalopathy; noHE, non-hepatic encephalopathy; HC, health control.

The results showed that the node disjointness score of MHE patients was significantly higher than that of noHE patients at some specific brain regions, which mainly fell in Frontal_ Sup_Medial_L/R, Frontal_Mid_Orb_R, Frontal_Sup_L/R and Cingulum_Mid_L (Figure 3A). MHE patients were significantly lower than noHE patients only in the area of the Temporal_Mid_R. It can be seen that compared with noHE patients, MHE patients have more frequent single-node switching rate in regions in DMN and FPN and less frequent single-node switching rate in region of VAN.

In order to better understand the pathogenesis, we also conducted a control analysis between MHE patients and the HC group. The results showed a significant difference in Frontal_Inf_Tri_L, Frontal_Sup_Medial_L, Parietal_Inf_R, Cuneus_L, Frontal_Sup_R, Precuneus_R areas (Figure 3B). It can be seen that MHE patients had a higher single-node switching rate than HC in most regions, except in the Frontal_Inf_Tri_L region. The difference regions were located in DMN, FPN, ventral attention network (VAN), VN and SSHN.

Meanwhile, the noHE patients and the HC group were compared. The disjointness of noHE patients was significantly higher than HC in Insula_R, Precuneus_L, Frontal_Mid_L, but lower in Frontal_Mid_R (Figure 3C). The difference nodes mainly fell into DMN, FPN, SN and sensory/somatomotor hand network (SSHN).

In addition, we analyzed the effect of disease on flexibility and cohesion metrics, but no significant group difference was found at *p* < 0.05 after corrections for multiple comparisons.

### Correlation Results Between Dynamic Nodal Metrics and Clinical Scores

**FIGURE 4:**
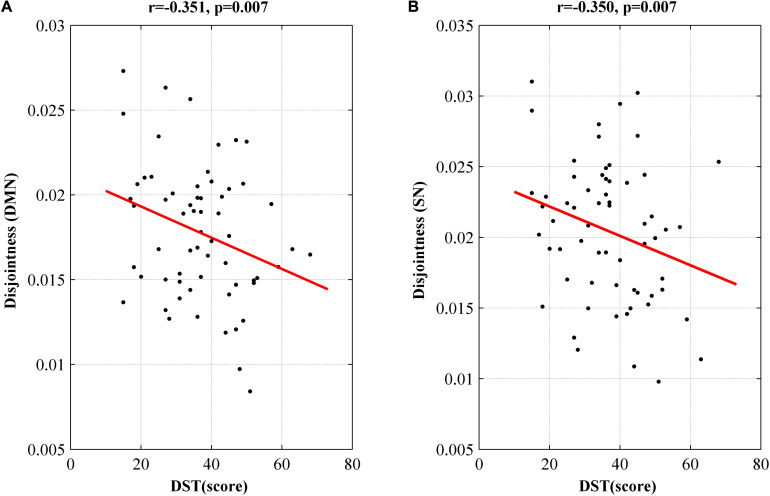
Networks showing significant correlation of disjointness metric with DST score in DMN **(A)** and SN **(B)**. DMN, default model network; SN, salience network; DST, digit symbol test; MHE, minimal hepatic encephalopathy.

At the network level, we observed a significant negative correlation between disjointness and DST score in DMN and SN (*p* < 0.05, *r* > −0.4) (Figure 4). We didn’t find correlation between cohesion or flexibility and all the clinical testing scores.

At the node level, there are much more nodes showing correlation between disjointness and scores. These nodes are listed in Supplementary Table 1. Some correlative nodes were also found between cohesion or flexibility and clinical scores, but it should be noticed that these nodes were mostly similar. This suggests that the community flexibility is most likely caused by nodes partition change in pairs. The higher correlation in the disjointness metrics also indicates that community switching of nodes in the disease population were more likely changed individually.

Validation analysis to check for robustness of correlation results with different modularity parameters or different window parameters are provided in the Supplementary Information (Supplementary Tables 1–6).

### Classification Results of MHE From noHE

Considering node disjointness is the unique metric that can reveal difference between MHE and noHE among the three dynamic network properties, and it also shows correlation with neuropsychological scores in patient groups, we take this metric as discriminant features for identification MHE from noHE. The accuracy, sensitivity and specificity of SVM classifier with radial basis function (RBF) kernel were 88.71, 92.31, and 83.33%, respectively. The permutation tests reveal a significance level of *p* < 0.001 for accuracy, which suggests that the identification accuracy was significantly higher than chance level, indicating the effectiveness of the identification model.

The classification results of MHE from noHE based on difference window size are shown in Table 5. All results achieved a cross-validated classification accuracy above 83.87%, indicating the robustness of the selected window length. On the other hand, we obtained optimal window length of 45TR (112.5 s), basically consistent with the result that the optimal classification accuracy was obtained around the window length of 110s (Liu et al., 2017).

**TABLE 5:** The classification results with different window length.

	Window length (40TR)	Window length (45TR)	Window length (50TR)	Window length (55TR)
ACC	85.48%	**88.71%**	85.48%	83.87%
SEN	92%	**92.31%**	88.89%	85.71%
SPE	81.08%	**83.33%**	84.38%	82.35%

**FIGURE 5:**
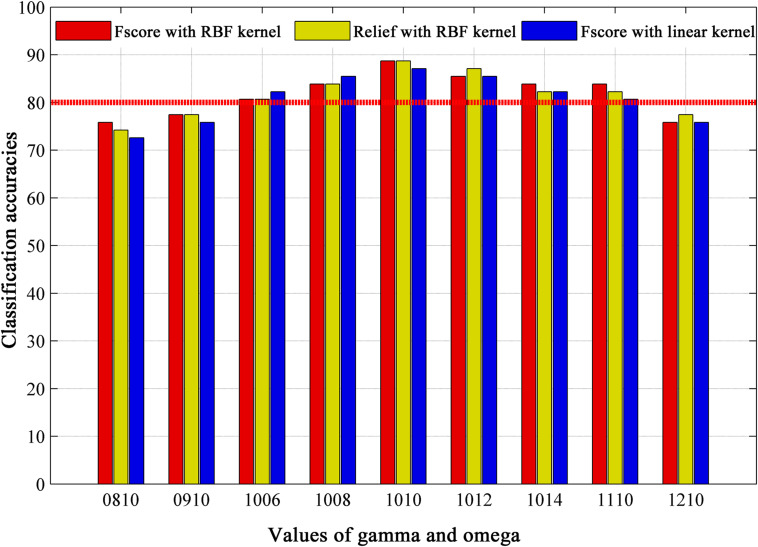
The classification results based on different module partition parameters (gamma, omega), feature selection methods (Fscore and Relief), and kernel functions of classifier (RBF kernel and linear kernel), where omega is spatial resolution parameter, and gamma is temporal resolution parameter in multi-layer community structure calculation. Values of gamma and omega = 0810 mean gamma = 0.8, omega = 1.0. RBF = radial basis function.

Figure 5 displays the classification accuracies based on different feature selection method, SVM kernel function and module partition parameters. For a fixed value of gamma (gamma = 1.0), we found a stable accuracy above 80% in omega value range between 0.6 and 1.4. When performing analysis in other topological scales (gamma = 0.8, gamma = 0.9, gamma = 1.1 with omega = 1.0), we observed a slightly accuracy decrease. We also paid attention to the impact of feature selection on classification accuracy. The commonly used feature selection method “relief” was used as a comparison, the results show almost no difference in classification accuracy. Altogether, the best accuracy is obtained at 88.71% with parameters of gamma = 1.0, omega = 1.0, *F*-score method and RBF kernel in SVM.

**FIGURE 6:**
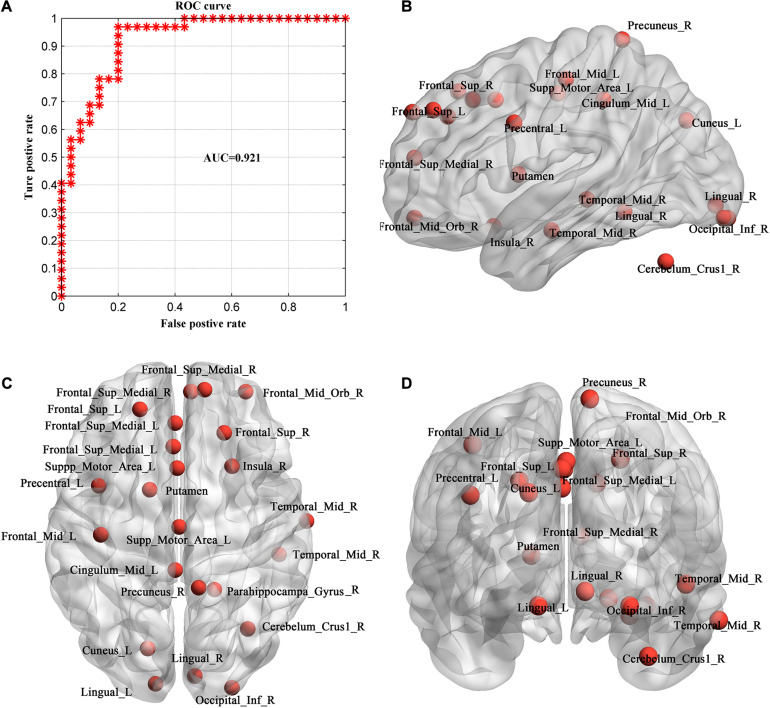
Evaluation of individual MHE discrimination classifier. **(A)** ROC curve of the classifier. **(B–D)** The consistent discriminant nodes in SVM classification showing in sagittal **(B)**, axial **(C)** and coronal planes **(D)**. MHE, minimal hepatic encephalopathy. ROC, receiver operating characteristic; AUC, = area under ROC curve.

The ROC curve using each subject’s classification score as a threshold are shown in Figure 6A. The area under the ROC curve (AUC) of the proposed method was 0.921, indicating an excellent discriminative power.

Because we used LOOCV strategy to train the model, features are different in different loops. The frequently occurred discriminative feature nodes in all loops were displayed in Figures 6B–D, which included Occipital_Inf_R, Precuneus_R, Cingulum_Mid_L, Frontal_Sup_L/R, Lingual_L/R, Frontal_ Sup_Medial_L/R, Cuneus_L, Frontal_Mid_Orb_R, Precentral_L, Insula_R, Temporal_Mid_R, Putamen, Supp_Motor_Area_L, Cerebelum_Crus1_R.

## Discussion

Cognitive decline in cirrhotic patient with occurrence of MHE has been largely acknowledged (Damulin, 2018), but the current clinical diagnosis based on neuropsychological cognitive tests is easily mixed by sex, age and education level. In this study, we aimed to investigate the biomark of MHE based on brain imaging data. From a view of dynamic brain graph analysis, we took the whole brain functional connectivity matrix as a graph and applied multi-layer modular algorithm to analyze the dynamic reorganization of the modules in brain graph of MHE patients. Connection-stability profile and nodal affiliation changes were used to describe the dynamic graph properties of the brain network. By comparing these metrics among groups, we found that in the connection-stability matrix, MHE and HC showed significant differences within specific networks, but there was no statistical significance between other groups at network level. In terms of nodal dynamic metrics, MHE and noHE showed significant differences merely in node disjointness. Further, we observed node disjointness in patient groups were correlated with neuropsychological scales. In order to test the discriminative power in identification MHE from noHE, we used SVM to classify the two groups based on node disjointness metric. Consistent discriminant nodes were identified contributing to a better explanation of dysfunctional mechanisms. The high classification accuracy of 88.71% also suggest the effectiveness of the dynamic brain functional metric in identification of individual MHE.

In our study, the connection-stability matrix quantifies the relative stability of node pairs in a dynamic process. A higher value means less flexibility of the node pairs. From the matrix profile of the three groups, it can be seen that the obtained connection-stability matrix in healthy control group showed obvious modularity, which was similar to Power’s 14 functional network partition results (Power et al., 2013). This suggests that the modular partition results in the present study are confidential and are consistent with brain functional network organization patterns. Compared with healthy control group, the average connection stability within SSMN, CON, DMN, and FPN were significantly reduced in MHE patients (Table 3). This is consistent with previous studies reporting that the abnormal network strength in these networks (Chen et al., 2014; Jao et al., 2015; Cheng et al., 2018). Because the SSMN is important for motor control function (Matyas et al., 2010), the CON, DMN and FPN are responsible for execute control function, attention, and working memory that are important in daily activities (Fox et al., 2005; Matyas et al., 2010), the abnormal connection stability with these networks may explain the impairs in daily functioning, driving performance, work capability and learning ability in MHE patients (Agrawal et al., 2015). There was no significant difference in the average connection-stability matrix between MHE and noHE at the network level, but at the node level, nodal disjointness difference was found between the two groups, indicating that conversion from noHE to MHE is represented by some nodal dysfunction within networks but not the whole network dysfunction.

Further analysis of three node properties in comparison of MHE with noHE indicates that significant differences were found merely in node disjointness metric. The regions that showed abnormal node disjointness were in middle temporal region, superior medial and lateral regions, orbital frontal region, middle cingulate cortex, and they mainly occupied the VAN, FPN and DMN, which are important for attention allocation and execution control functions that have decline in MHE patients (Rosazza and Minati, 2011; Zhang et al., 2014a). These damaged areas of MHE were also reported in previous studies (Chen et al., 2016a; Zhang et al., 2018a). Comparison of the three groups together, we can see in cirrhosis with no HE, the affected regions were located in both primary network (SSHN) and high level cognitive networks (SN, FPN, and DMN). When progression from noHE to MHE, the dysfunctional nodes were mainly distributed in the three high-level core cognitive networks (VAN, FPN, and DMN), demonstrating that occurrence of MHE is related to core cognitive dysfunction. On flexibility and cohesion, we found no significant differences, suggesting that brain impairment in MHE patients might be caused by the abnormal proportion of nodes independently switching network affiliation but not the nodes that change network affiliation with other nodes together. The results also show that single-node switching may be more frequent in brain regions in MHE patients than in noHE patients. This may lead to cognition-related dysfunction in MHE patients.

The cognitive deficit of all cirrhotic patients was evaluated using DST and NCT-A scores in this study as recommended (Weissenborn et al., 2001; Li et al., 2013), in which DST evaluates cognitive function of attention and visual memory (Weissenborn et al., 2001; Zhang et al., 2017a). Correlation analysis showed that DST scores were positively correlated with the average connection stability scores of DMN, VN, FPN, SN, subcortical network and had negative correlations with the nodal disjointness values of the DMN and SN. Theoretically, a higher flexibility of the nodes corresponds to a lower average connection stability, and thus consistency in the two correlation analyses were found in DMN and SN. Both of the two networks are important for attention and working memory (Pessoa et al., 2002; Silk et al., 2010). DMN collectively comprises an integrated system for different aspects of self-referential mental processes and it is de-actived in a cognition-demanding task (Raichle et al., 2001). SN is important for detection and mapping of salient external inputs and internal brain events, and it drives the switching between default mode and central executive networks in a triple-network model underlying the high level cognitive function (Menon, 2011). Therefore, correlation between the node disjointness of the two networks and the DST scores indicates that increased single-node switching rate in DMN and SN may be responsible for decline in high level cognitive functions such as attention and working memory that are important in the cirrhotic patients’ daily life.

Since we found significant differences between MHE and noHE merely in the node disjointness at network level and unique correlation with neuropsychological scores was also obtained in this metric, we used node disjointness to evaluate the discriminative power of classifier. The experiment results show its effectiveness in identification of individual MHE from noHE with an accuracy of 88.71%. Comparison analyses show that the spatial resolution parameter of omega had a greater impact on the results than temporal resolution parameter gamma. The best result was obtained when we chose the default values gamma = 1.0 and omega = 1.0 as the module partition parameters. With the best omega of 1.0, each network module was functionally explainable (see Figure 2). In respect of the impact of time window size, it is not a parameter that affects our main results. Results showed that the accuracy was highest when the time window length was 45TR (112.5 s), which is consistent with a previous study reported that classification accuracy was highest when the time window length was around 110s (Liu et al., 2017). With the comparisons of different combinations of feature selection method and classifiers, the best accuracy was obtained with F-score method and SVM with RBF kernel. In general, by validation on different parameters, our results showed a good discriminative power at individual level. Furthermore, by comparing the discriminative nodes obtained from individual diagnosis with the results from group statistical analysis, we can see not only overlapping regions, but also other regions were found in the discriminative analysis. This is because the discriminative analysis can grasp the whole pattern information in a multivariate way than the univariate group statistical analysis, demonstrating that combination of the dynamic features with machine learning model can provide more information.

However, some factors may limit the generalization of our findings in clinical application. First, because of the difficulty in patient recruitment, the sample size is small in this study, which may bias the result. Large samples can improve statistical power and reduce overfitting in the identification model. In the future, we will verify the identification model by both internal and external validations with lager sample size to provide a stable performance. Other more complex models like deep neural networks that can grasp deep and complex MHE characteristics can also be tried with larger dataset to provide better accuracy. Second, the laboratory test data was not used in the identification model. It could be combined with imaging data together into the identification model, which may provide a better identification accuracy. Third, different medications among patients may affect the findings in this study. In our study, lactulose was used in 19 patients to improve feces excretion function, and they took 5–10 g lactulose three times a day. Antibiotics was used in some patients who had infections such as spontaneous bacterial peritonitis, pulmonary infection, which may bias the findings because antibiotics may induce changes in synaptic plasticity and neural cell growth (Heiss and Olofsson, 2019). In addition, the smoking history may also affect the result, which should be considered in the future studies. Last, laboratory test data are needed for healthy controls to fully exclude the possible liver disease.

## Conclusion

Previous studies mainly used static analysis features to study MHE, we used the multi-layer modular algorithm based dynamic functional connectivity features to study and identify MHE from noHE. The core of this method is to extract metrics describing network connection-stability and dynamic nodal affiliation based on dynamic module partition results. It is found that compared with HC, MHE showed differences in both primary sensory network module and high level cognitive networks, consistent with the symptom of decline in driving ability, daily functioning, learning and working performance. When progression from noHE to MHE, the cognitive impairments were mainly in higher cognitive networks of DMN and SN by disrupting network organization in a way of frequent single-node disjointness, which is associated with the neuropsychological score. Based on the node disjointness property, the individual discriminative model showed a diagnosis accuracy of 88.71%. Taken together, the results in this study suggest that dynamic graph analysis can reveal the brain network evolvement patterns from noHE to MHE, help us understand the dysfunction of the brain in a fine scale, and provide powerful features for the individual diagnosis. In the future, larger samples of patients with no mixture of interference factors are needed to verify the findings and further optimize the model to improve the identification accuracy. We can further investigate the clinical features of MHE on electroencephalo-graph (EEG). EEG features and the clinical data from laboratory test can also be incorporated into the machine learning model to develop a tool that can be used with lower cost and rural areas without fMRI.

## Data Availability Statement

The original contributions presented in the study are included in the article/Supplementary Material, further inquiries can be directed to the corresponding author/s.

## Ethics Statement

The studies involving human participants were reviewed and approved by Medical Research Ethics Committee of Tianjin First Central Hospital. The patients/participants provided their written informed consent to participate in this study.

## Author Contributions

YC and WS designed the experiment, recruited the participants, and revised the manuscript. GZ proposed the data analysis method, performed data analysis, and wrote and revised the manuscript. YL performed data analysis and wrote the original manuscript. XZ and LH contributed to data collection. All authors contributed to the article and approved the submitted version.

## Conflict of Interest

The authors declare that the research was conducted in the absence of any commercial or financial relationships that could be construed as a potential conflict of interest.

## Footnotes

1 https://www.nitrc.org/projects/gretna/

2 http://www.csie.ntu.edu.tw/∼cjlin/libsvm/

## Abbreviations

Cerebelum_Crus1_R: right cerebellum crus 1 region
Cingulum_Mid_L/R: left or right middle cingulum region
Cuneus_L: left cuneus region
Frontal_Inf_Tri_L: left inferior triangle frontal region
Frontal_Mid_Orb_L/R: left or right orbital middle frontal region
Frontal_Sup_L/R: left or right superior frontal region
Frontal_Sup_Medial_L: left superior medial frontal region
Frontal_Sup_Medial_L/R: left or right superior medial frontal region
Insula_R: right insular region
Lingual_L/R: left or right lingual region
Occipital_Inf_R: right inferior occipital region
Parietal_Inf_R: right inferior parietal region
Precentral_L: left precentral gyrus
Precuneus_L/R: left or right precuneus
Supp_Motor_Area_L: left supplemental motor area
Temporal_Mid_R: right middle temporal region.
