# Supplementary material for: Identifying Mild Hepatic Encephalopathy Based on Multi-Layer Modular Algorithm and Machine Learning
Source: Front Neurosci. 2021 Jan 11;14:627062. doi: 10.3389/fnins.2020.627062 (PMC7829502; doi:10.3389/fnins.2020.627062)
Supplement: Supplementary file 1 [file Table_1.DOCX]

**Supplemental Information**

**Correlation between dynamic nodal metrics and clinical scores at node level**

The correlation between node disjointness and clinical scores at node level is shown in Table S1. To analyze the robustness of our analysis with window parameters, we repeated the correction analysis with window sizes of 40TR, 45TR, 50TR, 55TR. We obtained similar correlative nodes between disjointness and blood ammonia, neuropsychological tests score (Table S1). And correlative network between disjointness and digit symbol test score mainly covered Default_mode Network, Memory_retrieval Network, Visual Network, Fronto-parietal_Task_Control Network, Salience Network and Subcortical (Table S2).

We further demonstrate the robustness of our correlation results by slightly varying the modularity parameters. At the gamma=1.0 considered, we found a convergence to similar significant correlative nodes and networks between disjointness and blood ammonia, neuropsychological tests score in parameter range between 0.6-1.4 for omega values. The similar results also occurred with omega=1.0 and gamma values range between 0.8-1.2 (Table S3-S6).

**Table S1.** The correlation between disjointness and Blood Ammonia, NCT-A, DST within different time window lengths. In order to better detect the robustness, nodes with p<0.05 were represented by node number.

| Window length | Blood Ammonia | NCT-A | DST |
| --- | --- | --- | --- |
| win40 | 17 74 96 105 112 149 173 190 202 205 228 263 | 28 73 74 84 85 94 97 104 105 112 160 185 187 199 202 214 | 8 17 36 74 84 85 87 100 104 105 109 110 112 148 150 160 163 164 171 177 185 190 195 197 199 202 214 216 218 219 228 260 |
| win45 | 17 22 112 149 173 184 190 205 228 262 263 | 73 84 85 94 97 104 105 112 181 185 202 228 247 | 17 36 84 85 97 101 104 105 112 113 116 150 160 163 164 171 177 185 190 195 197 202 214 216 218 219 228 244 258 260 |
| win50 | 32 62 84 105 112 149 179 184 190 262 263 | 17 84 85 94 97 102 104 105 112 117 137 160 181 190 219 227 228 | 17 22 36 84 85 97 101 104 105 112 116 119 121 122 137 153 160 164 165 166 171 184 185 190 197 213 217 219 228 260 |
| win55 | 17 32 62 84 105 112 149 179 184 190 262 263 | 17 84 85 94 97 102 104 105 112 117 121 137 160 181 190 219 227 228 | 17 22 36 84 85 97 101 104 105 112 116 119 121 122 137 153 160 164 165 166 171 184 185 190 197 213 214 216 217 219 228 260 |

**Table S2.** The significant correlated network (P < 0.05, ^*^ represents survival after FDR correction) between network average disjointness and digit symbol test score.

| Window length | win40 | win45 | win50 | win55 |
| --- | --- | --- | --- | --- |
| Network index | 7 9 10 11 | 6* 7 9 10 11* | 6* 7* 9* 10* 11* 12 | 6* 9 10* 11* |

**Table S3.** At the variable omega value, the significant correlated nodes (P< 0.05) between disjointness and blood ammonia, neuropsychological tests score.

| Gamma_  Omega | Blood Ammonia | NCT-A | DST |
| --- | --- | --- | --- |
| 10_06 | 32 74 105 161 263 | 15 85 94 97 104 105 112 137 160 202 | 17 36 74 84 85 97 101 104 105 112 113 116 121 134 150 160 163 164 171 177 185 190 193 195 197 199 202 213 214 216 218 219 228 260 263 |
| 10_08 | 32 74 105 149 161 190 228 263 | 2 15 85 94 97 104 105 112 160 202 203 | 17 36 74 84 85 97 101 104 105 112 113 116 121 146 150 160 163 164 171 185 190 193 195 197 199 202 214 216 218 219 228 260 263 |
| 10_10 | 17 22 112 149 173 184 190 205 228 262 263 | 73 84 85 94 97 104 105 112 181 185 202 228 247 | 17 36 84 85 97 101 104 105 112 113 116 150 160 163 164 171 177 185 190 195 197 202 214 216 218 219 228 244 258 260 |
| 10_12 | 74 105 110 112 149 161 173 190 228 263 | 15 84 85 94 97 102 104 105 112 115 137 181 202 214 227 228 247 | 17 36 42 60 74 84 85 97 104 105 109 112 113 150 160 163 164 166 171 173 179 185 190 195 197 202 208 214 215 216 219 228 254 258 260 |
| 10_14 | 32 74 105 149 161 171 173 190 228 263 | 15 84 85 97 102 104 112 153 181 202 214 227 228 | 17 22 36 60 84 85 93 97 101 104 105 112 113 146 150 153 160 163 164 166 171 173 177 185 190 193 194 195 197 202 204 214 215 216 218 219 227 228 258 260 263 |

Gamma_Omega is modularity parameter, e.g. The value 10_08 represents gamma=1.0 and omega=0.8; NCT-A = Number Connectivity Test A; DST= Digit Symbol Test.

**Table S4.** The significant correlated network (P < 0.05, ^*^ represents survival after FDR correction) between network average disjointness and digit symbol test score.

| Gamma_Omega | 10_06 | 10_08 | 10_10 | 10_12 | 10_14 |
| --- | --- | --- | --- | --- | --- |
| Network index | 6* 7* 9 10 11* 12 | 6* 7* 9* 10* 11* 12 14 | 6* 7* 9* 10* 11* 12 | 6* 9* 10* 11* 14 | 6* 7 9* 10* 11* 14 |

Gamma_Omega is modularity parameter, e.g. The value 10_08 represents gamma=1.0 and omega=0.8.

**Table S5.** At the variable gamma value，the significantly correlative nodes (P< 0.05) between disjointness and blood ammonia, neuropsychological tests score.

| Gamma_  Omega | Blood Ammonia | NCT-A | DST |
| --- | --- | --- | --- |
| 08_10 | 32 48 89 112 122 143 149 155 173 221 263 | 73 74 97 115 117 137 140 153 160 181 184 214 219 227 | 17 22 25 36 42 49 60 74 104 112 113 116 122 127 146 147 150 153 160 164 165 171 184 190 191 202 214 215 216 219 227 258 263 |
| 09_10 | 17 22 32 89 112 122 147 149 173 190 197 220 228 263 | 46 85 94 97 112 115 117 140 153 160 185 214 227 228 247 | 17 21 22 36 42 60 74 94 104 112 116 146 147 150 157 160 162 163 164 165 169 171 185 190 195 197 202 214 215 216 219 228 258 260 |
| 10_10 | 17 22 112 149 173 184 190 205 228 262 263 | 73 84 85 94 97 104 105 112 181 185 202 228 247 | 17 36 84 85 97 101 104 105 112 113 116 150 160 163 164 171 177 185 190 195 197 202 214 216 218 219 228 244 258 260 |
| 11_10 | 112 134 149 161 190 193 197 228 262 263 | 73 85 97 102 104 105 112 137 160 181 247 | 17 36 84 85 97 101 102 104 105 109 110 112 113 150 160 163 164 169 171 185 190 195 197 199 202 214 218 219 228 244 258 260 263 |
| 12_10 | 17 36 105 112 123 134 161 179 193 217 263 | 15 73 85 97 102 104 105 112 137 181 247 | 17 36 84 97 101 102 104 105 109 110 112 121 140 150 163 167 169 171 185 195 197 199 202 213 214 215 218 219 228 244 263 |

Gamma_Omega is modularity parameter, e.g. The value 10_08 represents gamma=1.0 and omega=0.8; NCT-A = Number Connectivity Test A; DST= Digit Symbol Test.

**Table S6.** The significant correlated network (P < 0.05, ^*^ represents survival after FDR correction) between network average disjointness and digit symbol test score.

| Gamma_Omega | 08_10 | 09_10 | 10_10 | 11_10 | 12_10 |
| --- | --- | --- | --- | --- | --- |
| Network index | 7 9 10 11 | 6 7 9 10 11 | 6* 7* 9* 10* 11* 12 | 6* 9 10* 11* | 6* 9 10* 11* |

Gamma_Omega is modularity parameter, e.g. The value 08_10 represents gamma=1.0 and omega=0.8.
